# Dynamic Contrast-Enhanced Magnetic Resonance Lymphangiography for Diagnosis and Treatment of Chylopericardium After Cardiac Transplantation

**DOI:** 10.1016/j.jaccas.2024.102386

**Published:** 2024-06-14

**Authors:** Jorge Landazabal, Eduardo Antonio Villa-Pallares, Yoav Dori, Néstor Sandoval, Jhon Ramírez, Oscar Mauricio Pérez-Fernández, Gabriel Caviedes, Julián Forero, Carlos-Eduardo Guerrero-Chalela, Laura Acosta Izquierdo

**Affiliations:** a Cardiology Department, Fundación Cardioinfantil–Instituto Cardiología, Bogotá, Colombia; b School of Medicine and Health Sciences, Universidad del Rosario, Bogotá, Colombia; c Division of Cardiology, Department of Pediatrics, Children’s Hospital of Philadelphia and University of Pennsylvania Perelman School of Medicine, Philadelphia, Pennsylvania, USA; d Congenital Heart Institute, Fundación Cardioinfantil–Instituto Cardiología, Bogotá, Colombia; e Radiology Department, Fundación Cardioinfantil–Instituto Cardiología, Bogotá, Colombia

**Keywords:** chylopericardium, heart transplant, lymphangiography, magnetic resonance

## Abstract

Chylopericardium is a rare complication after cardiac transplantation. We report a case of a 69-year-old woman with persistent chylopericardium after a heart transplantation due to Chagas disease. Failure of conservative treatment led to dynamic contrast-enhanced magnetic resonance lymphangiography and percutaneous radiologic intervention of the lymphatic leakage and symptoms resolution.

## Graphical Abstract

**Figure:**
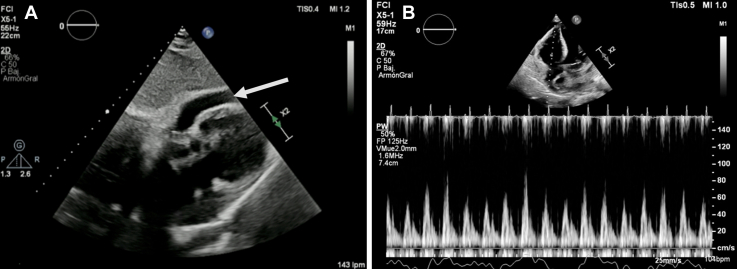

## History of Presentation

**Figure 1:**
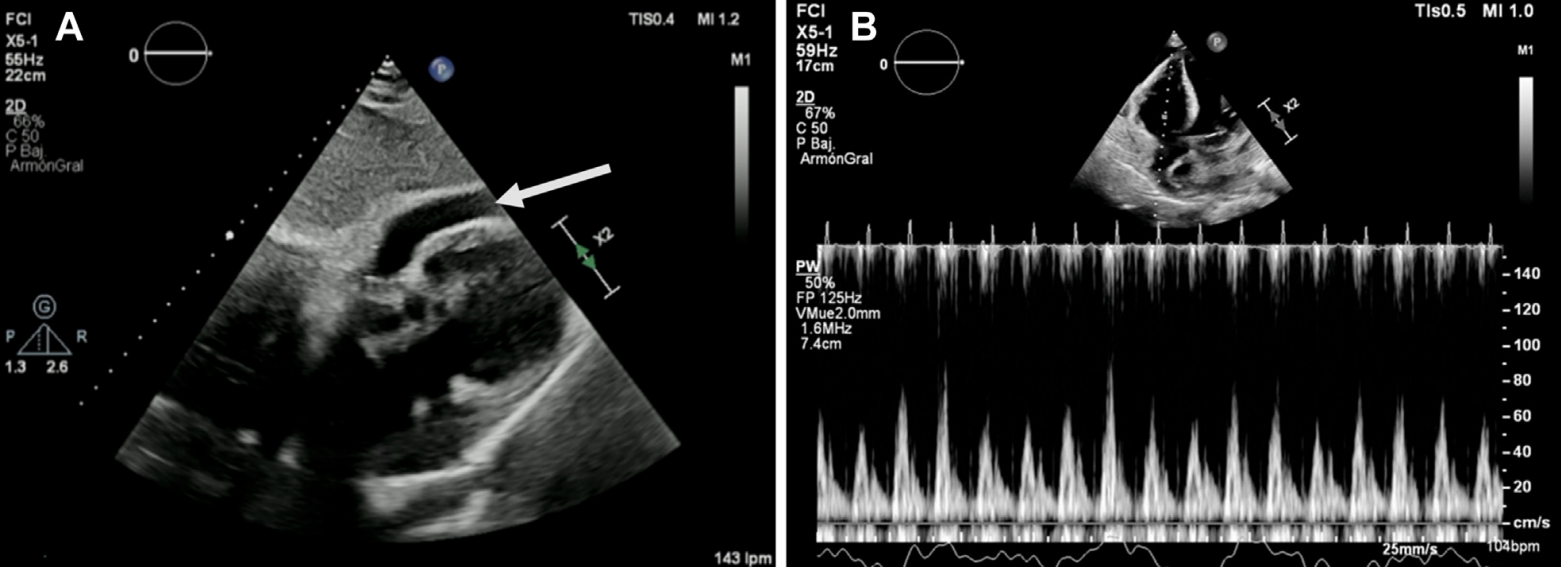
Transthoracic Echocardiogram (A) Moderate pericardial effusion (arrow) and (B) transtricuspid doppler evaluation showing marked inflow variability suggestive of increased intrapericardial pressures.

A 69-year-old woman with history of shortness of breath, orthopnea, and lower extremities swelling, had multiple hospitalizations in the last 3 months due to similar symptoms. On presentation, her blood pressure was 100/70 mm Hg. She was tachycardic on examination with a heart rate of 110 beats/min, regular rhythm, an oxygen saturation of 97% on room air, and a temperature of 36.7°C. Physical examination revealed increased jugular venous pressure and muffled heart sounds with clear lungs. Initial work-up included a chest x-ray that showed an enlarged cardiac silhouette with clear lungs, status poststernotomy, and an abandoned lead in the left subclavian vein. A transthoracic echocardiogram revealed moderate pericardial effusion with signs of increased pericardial pressures (Figures 1A and 1B).

**Learning Objectives**

• To recognize the potential occurrence of Chylopericardium after cardiac transplantation, particularly in the presence of traumatic surgical lesions, toward precise accurate identification and management.
• To demonstrate the importance of DCMRL to identify and assess lymphatic leakage, guiding effective treatment approaches.
• To promote the use of percutaneous lymphatic interventions in cardiac patients because they are a less morbid treatment option for patients with traumatic chylopericardium.

## Medical History

She underwent a heart transplantation 6 months earlier due to Chagas disease, with primary graft dysfunction and 2R cell rejection at 3 months post-transplantation, treated aggressively with immunosuppressive therapy. Notably, an implantable cardioverter-defibrillator (ICD) lead was left abandoned in the left subclavian vein. Mild pericardial effusion in follow-up was considered related to transplant rejection.

## Differential Diagnosis

In the context of our case, the differential diagnosis included postpericardiotomy syndrome, graft rejection after the recent heart transplantation, infective pericarditis, iatrogenic chylopericardium, or iatrogenic pericardial effusion related to biopsy.

## Investigations

**Figure 2:**
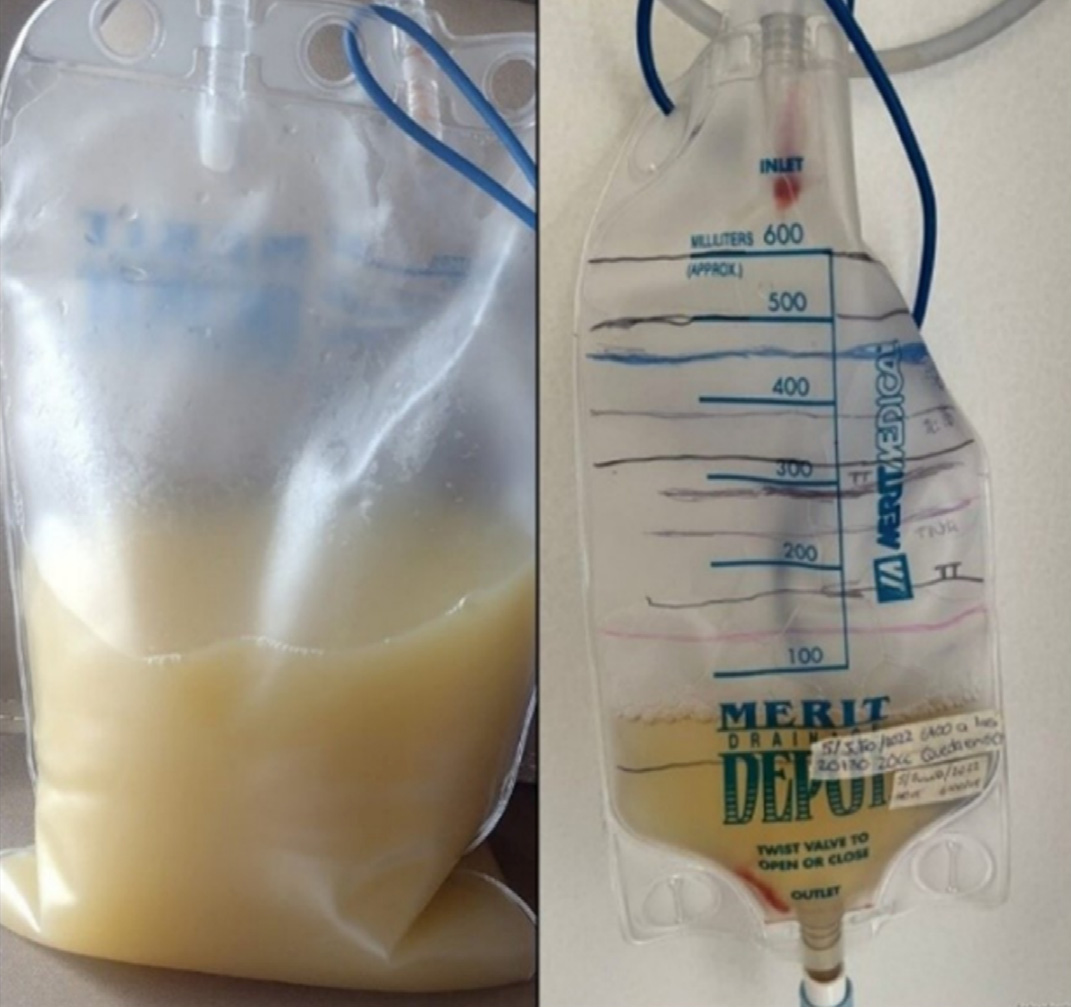
Macroscopic Characteristics Macroscopic characteristics of chylous pericardial fluid.

A pericardiocentesis obtained 1,000 mL of milky pericardial fluid (Figure 2). The analysis showed a triglyceride concentration of 313 mg/dL with a predominant lymphocytic cell count (92%) (Table 1). Ziehl-Neelsen stain and adenosine deaminase tests were negative, with no bacterial growth on cultures, which is overall compatible with chylopericardium. Additional examinations included a right-sided heart catheterization, complemented by superior and inferior vena cava venography, to evaluate potential obstruction of venous flow at the thoracic duct drainage by the abandoned ICD lead. The absence of significant stenosis and normal caval pressures effectively dismisses the possibility of venous hypertension. It remains plausible that an attempt to extract the lead during heart transplantation could have incurred damage to the thoracic duct drainage site. Conservative management was chosen, including parenteral nutrition therapy with medium-chain fatty acids, octreotide administration, and continuous pericardial catheter drainage. After treatment failure, consideration was given to a thoracoscopic approach; however, this option was dismissed because the presumed level of leakage could not be effectively addressed through such a method. Consequently, percutaneous management was chosen.

**Table 1:** Pericardial Fluid Study

Test	Result
Cytochemical	
Proteins	5.1 g/dL
Lactate dehydrogenase	161 U/L
Leukocytes	197 cells/mL
Lymphocytes	92%
Triglycerides	313 mg/dL
Cultures and infection evaluation	
Leukocyte reaction	Abundant
Bacterial and fungi culture	Negative
Ziehl-Neelsen stain	Negative
Adenosine deaminase	13.1 U/L
Mycobacterium culture	Negative

## Management

**Figure 3:**
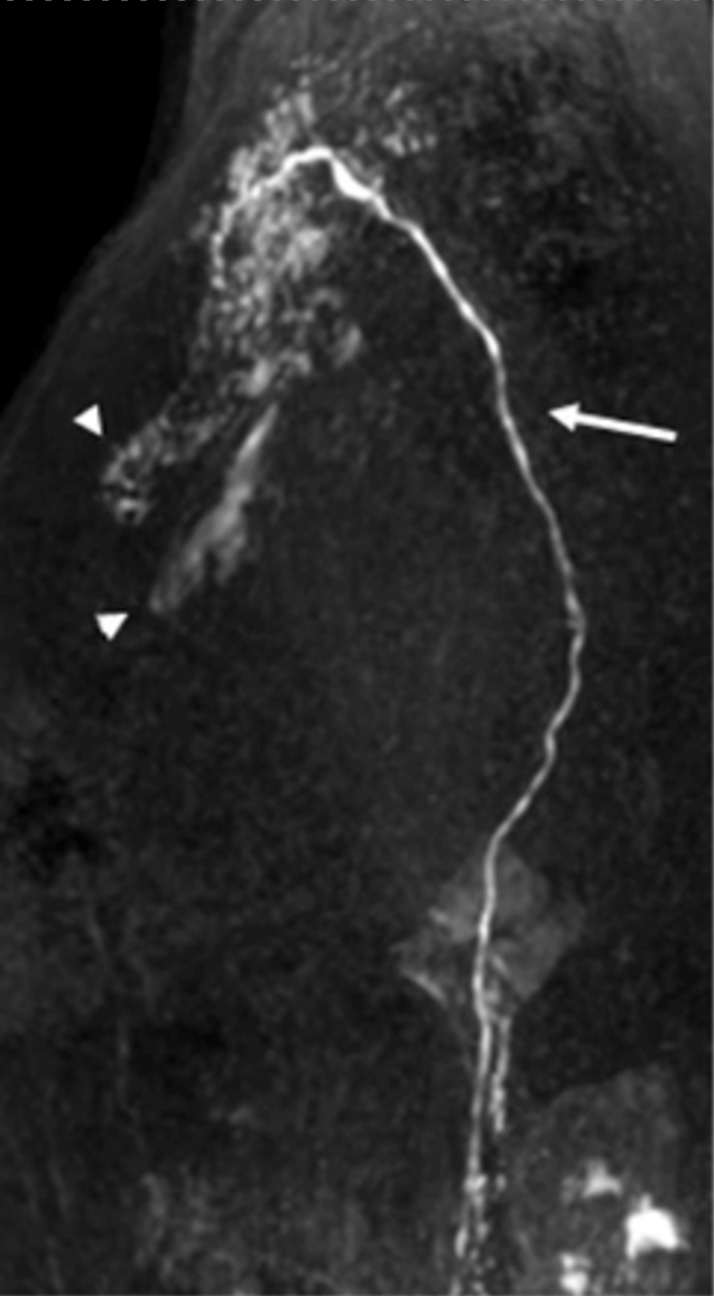
DCMRL DCMRL, sagittal reconstruction in a maximal intensity projection showing the thoracic duct (arrow), chylous reflux, and leakage into the perivascular and pericardial space (arrowheads). DCMRL = dynamic contrast-enhanced magnetic resonance lymphangiography

**Figure 4:**
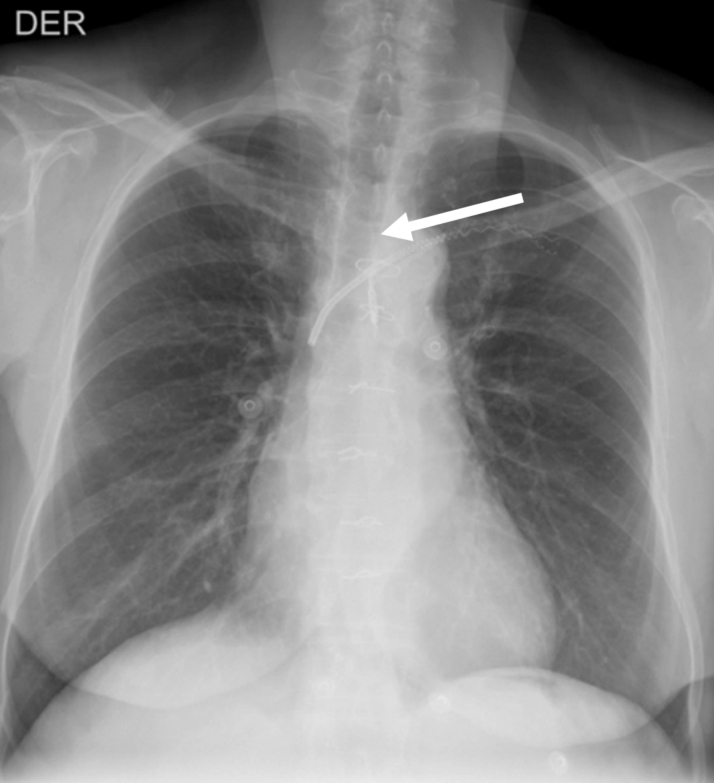
Postero-Anterior View Postero-anterior view of the chest, 3 months after the thoracic duct embolized, shows no pericardial effusion recurrence. Note embolization coils projected over the superior aspect of the thoracic duct (arrow).

With international proctoring, a dynamic contrast-enhanced magnetic resonance lymphangiography (DCMRL) was performed by cannulating bilateral inguinal lymph nodes. The procedure involved a standard time-resolved angiography with phases every 30 s after intranodal contrast injection with a total scan duration of 30 min. At 20 min, the DCMRL showed chylous reflux and leakage in to the perivascular and pericardial space, confirming a traumatic chylopericardium (Figure 3). Subsequently, the patient was transferred to the interventional radiology laboratory, where a direct lymphangiography was performed through the same lymphatic approach, with a successful embolization of the thoracic duct leakage with embolization coils and glue (Figure 4).

## Discussion

Chylopericardium is a rare clinical condition characterized by accumulated chylous fluid in the pericardial space. Etiologic causes in order of frequency are idiopathic in 56% of cases and iatrogenic after cardiac surgery in 9% of cases, of which 3% are related to orthotropic cardiac transplantation.^1^ Other etiologic causes involve malignant lymphatic system diseases, postradiation, superior vena cava syndrome, and blunt trauma.^2^ Treatment involves repeated pericardial drainage, conservative management with a medium triglycerides chain diet, and total parental nutrition. ^1^ However, conservative treatment is only effective in 40%-50% of cases.^1^^,^^2^ Therefore, in many cases, intervention over the thoracic duct is considered mandatory in patients who do not respond to conservative therapy, described as daily drainage of 500 mL/d over 7 days or nutritional complications.

Surgical intervention historically was the standard of care in cases of failure of conservative therapy.^1^ However, with an enhanced evaluation of the lymphatic system using DCMRL, percutaneous lymphatic interventions have emerged as the treatment option for a broad spectrum of conditions affecting the lymphatic system function.^3^ Enhanced evaluation of the lymphatic system is essential for traumatic chylopericardium because studies have reported a 90% cure rate of percutaneous treatment when the thoracic duct can be adequately characterized with no mortality and minimal morbidity.^4^

Our institution’s initial experience with direct lymphangiography did not yield sufficient information about thoracic duct anatomy and accurate localization of lymphatic leakages. Moreover, the recent sternotomy and abandoned ICD lead added to the two-dimensional nature of the technique were thought to reduce the effectiveness of direct lymphangiography in this specific case. Consequently, considering that we were facing a high-flow lymphatic leakage, our team elected to use DCMRL to ascertain the location leakage presumed to be at the thoracic duct level, based on recent reports in the literature supporting DCMRL in similar scenarios.^5^^,^^6^

Percutaneous intervention of lymphatics disorders has gained momentum in patients with congenital heart disease where abnormal lymphatic perfusion syndromes develop, aiding the quality of life of patients suffering from lymphatics disorders and also suggesting being cost-effective in the treatment of these life-threatening conditions.^7^

Moreover, diagnosis based on DCMRL of lymphatic leakages is quickly evolving to become the method of choice for guiding interventions in patients with many lymphatic perfusion syndromes and traumatic lymphatic lesions after cardiac transplantation.^8^^,^^9^

In our patient, a surgical approach was considered too high a risk of affecting the graft anastomosis due to the recent heart transplantation. Moreover, the site of the leakage would mean a redo sternotomy. Therefore, a consensus was reached that a percutaneous approach seemed a safer treatment option.

Albeit to our knowledge, there is no direct comparison between a catheter-based approach and surgical approach, catheter-based interventions for thoracic traumatic chylous leakage have been shown to be clinically successful in a cohort of patients with traumatic thoracic duct chylous leaks with 98% success rate in the long-term follow-up (mean: 34 months).^10^

## Follow-up

**Figure 5:**
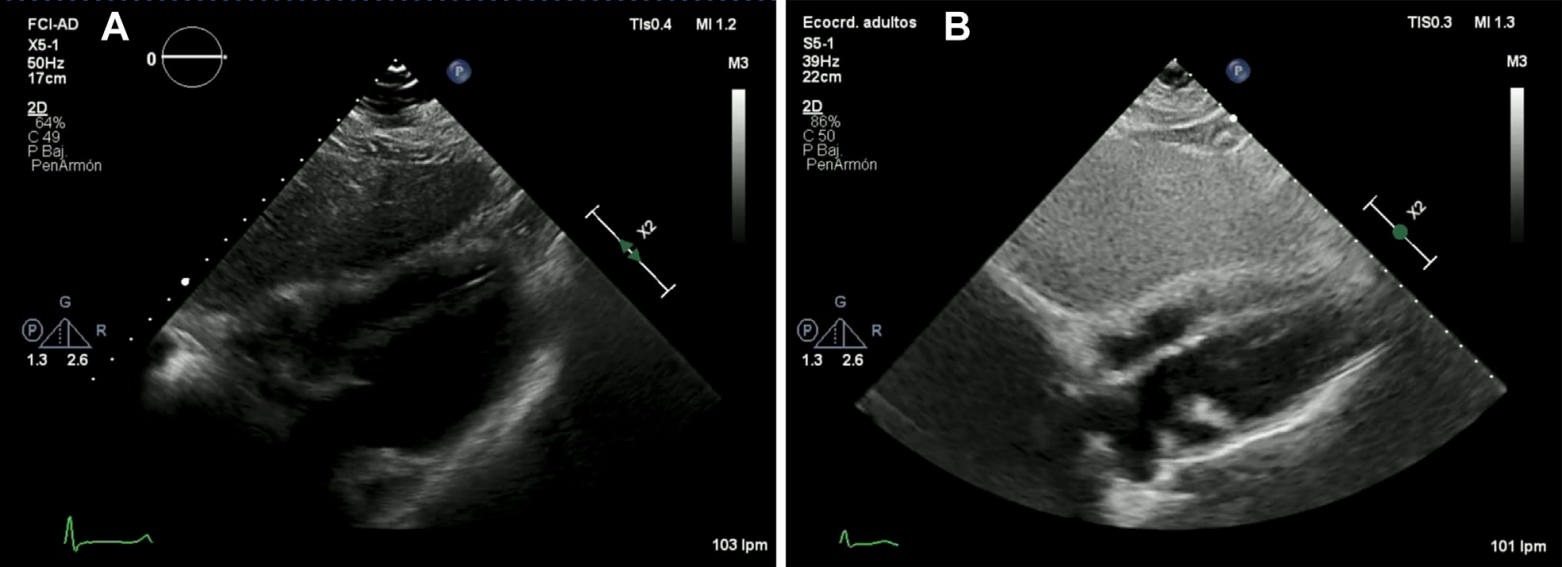
Follow-Up (A and B) Follow-up at 12 months indicates the resolution of pericardial effusion.

She remained asymptomatic during hospitalization with a gradual decrease in pericardial effusion. Midterm echocardiographic follow-up at 12 months shows minimal posterior pericardial effusion (Figure 5A and 5B) and resolution of symptoms.

## Conclusions

Chylopericardium is an uncommon complication after cardiac transplantation. Therefore, a high index of suspicion is needed. DCMRL has emerged as an essential tool to diagnose and guide treatment in patients with lymphatic leakages. When conservative treatment fails, the percutaneous approach to iatrogenic chylopericardium can be safely performed with excellent midterm results. Our case highlights the importance of a standardized diagnosis and treatment group approach for treating lymphatic disorders. This is the first report in Latin America on the use of DCMRL for guiding the treatment of lymphatic leakage.

## Funding Support and Author Disclosures

The authors have reported that they have no relationships relevant to the contents of this paper to disclose.
